# Privacy-Preserving Patient Similarity Learning in a Federated Environment: Development and Analysis

**DOI:** 10.2196/medinform.7744

**Published:** 2018-04-13

**Authors:** Junghye Lee, Jimeng Sun, Fei Wang, Shuang Wang, Chi-Hyuck Jun, Xiaoqian Jiang

**Affiliations:** ^1^ School of Management Engineering Ulsan National Institute of Science and Technology Ulsan Republic Of Korea; ^2^ Department of Biomedical Informatics University of California San Diego San Diego, CA United States; ^3^ Department of Industrial and Management Engineering Pohang University of Science and Technology Pohang Republic Of Korea; ^4^ College of Computing Georgia Institute of Technology Atlanta, GA United States; ^5^ Division of Health Informatics, Department of Healthcare Policy and Research Weill Cornell Medical College Cornell University New York City, NY United States

**Keywords:** privacy, federated environment, similarity learning, hashing, homomorphic encryption

## Abstract

**Background:**

There is an urgent need for the development of global analytic frameworks that can perform analyses in a privacy-preserving federated environment across multiple institutions without privacy leakage. A few studies on the topic of federated medical analysis have been conducted recently with the focus on several algorithms. However, none of them have solved similar patient matching, which is useful for applications such as cohort construction for cross-institution observational studies, disease surveillance, and clinical trials recruitment.

**Objective:**

The aim of this study was to present a privacy-preserving platform in a federated setting for patient similarity learning across institutions. Without sharing patient-level information, our model can find similar patients from one hospital to another.

**Methods:**

We proposed a federated patient hashing framework and developed a novel algorithm to learn context-specific hash codes to represent patients across institutions. The similarities between patients can be efficiently computed using the resulting hash codes of corresponding patients. To avoid security attack from reverse engineering on the model, we applied homomorphic encryption to patient similarity search in a federated setting.

**Results:**

We used sequential medical events extracted from the Multiparameter Intelligent Monitoring in Intensive Care-III database to evaluate the proposed algorithm in predicting the incidence of five diseases independently. Our algorithm achieved averaged area under the curves of 0.9154 and 0.8012 with balanced and imbalanced data, respectively, in *κ*-nearest neighbor with *κ*=3. We also confirmed privacy preservation in similarity search by using homomorphic encryption.

**Conclusions:**

The proposed algorithm can help search similar patients across institutions effectively to support federated data analysis in a privacy-preserving manner.

## Introduction

### Data-Driven Decision Making in Medical Fields

Electronic health records (EHRs) are becoming ubiquitous across almost all medical institutions. They provide insight into diagnoses [1-6], as well as prognoses [7-10] and can assist in the development of cost-effective treatment and management programs [8,11-14]. All kinds of data across institutions are being collected in EHRs, including diagnosis, medication, lab results, procedures, and clinical notes. In the recently announced precision medicine initiative, many more other types of data including omics data such as genomic and proteomic data and behavior data such as activity sensor data are being generated and collected by doctors and patients. As such rich and heterogeneous health data become available, the entire medical research and practice are shifting from the knowledge or guideline-driven approaches to the data or evidence-driven paradigm, where effective and efficient algorithms become the key for clinical research and practice.

### Limitations of Single-Institutional Studies

Previously, many biomedical studies were conducted within a single institution having limited EHR data because of the lack of federated data analysis framework and the institutional privacy concerns on data sharing. However, such an approach has many limitations. For example, it has been demonstrated that genome-wide association studies on EHR data often failed to discover known biomarkers from a single institution because of limited sample size [15,16]. To enable cross-institutional studies, many collaborative networks have been proposed, such as mini-sentinel [17], Observational Health Data Sciences and Informatics [18], National Patient-Centered Clinical Research Network [19], and i2b2 Shared Health Research Informatics Network [20]. These frameworks enable certain analyses (such as database queries with very specific inclusion or exclusion criteria) to be conducted efficiently in a federated manner. However, more sophisticated analyses such as predictive models [21] and context-specific patient similarity search [22] are still a challenge for most existing frameworks, as cross-institutional EHR data exchange is required to build such models, which is usually infeasible because of the institutional privacy and security concerns. There is an urgent need for the development of novel frameworks that can perform analyses in a privacy-preserving federated environment across multiple institutions. In this way, global analytic models can be built collectively without sharing raw EHR data. A few studies on the topic of federated clinical analysis [23-26] have been conducted recently with the focus on different algorithms. However, none of them have solved the problem of similar patient matching, which is important for many biomedical studies. Therefore, we plan to develop a privacy-preserving analytic platform that focuses on a suite of algorithmic challenges on patient similarity learning.

### Patient Similarity Learning

Patient similarity learning aims to develop computational algorithms for defining and locating clinically similar patients to a query patient under a specific clinical context [7,27-30]. The patient similarity search is very challenging because the raw EHR data is sparse, high-dimensional, and noisy, which makes finding an exact match among patients using EHR data almost impossible. Besides, patient similarity learning is often context-specific. For example, patient similarity measure for heart disease management can be very different from cancer management. The fundamental challenge is how we can perform effective context-specific patient similarity learning in a federated setting, which enables many different applications:

- Cohort construction: cross-institution observational studies are challenging but necessary as many studies require a large and specific patient cohort that does not exist within a single institution. To conduct such a study, an efficient similarity search needs to be conducted across institutions to identify the focused patient cohort.
- Disease surveillance: The Centers for Disease Control and Prevention monitors thousands of hospitals for potential epidemics. When a suspicious case is reported, there is a need to find similar cases across geographies.
- Clinical trial recruitment: pharmaceutical companies often need to spend significant amount of time and resources to identify targeted patients through many different clinical institutions. Ideally, they would like to be able to perform patient similarity search across all clinical institutions to identify where those relevant patients are. Then they can quickly focus on recruiting patients from the right clinical institutions.

Patient similarity learning involves two computational phases: (1) patient representation learning is to learn the context-specific representation of patients based on their EHR data. For example, patients may be given different representations in heart disease management versus cancer management and (2) patient similarity search is to find similar patients based on their corresponding representations. In a federated environment where multiple institutions exist, patient similarity learning has many unique challenges: (1) how to design an efficient but flexible patient representation that enables fast similarity search? (2) how to learn patient representation from heterogeneous data sources? and (3) how to preserve privacy while still allowing the computation of the patient representation and the search of similar patients across institutions?

### Research Objective

The main objective of this paper was to develop a privacy-preserving analytic platform for patient similarity learning in a distributed manner. We propose to learn context-specific binary hash codes to represent patients across institutions. The similarities between patients can be efficiently computed as the hamming distance using the resulting hash codes of corresponding patients; the hamming distance is defined to be the number of places where two binary codes differ. As patient data are heterogeneous from multiple sources such as diagnosis, medication, and lab results, we propose a multi-hash approach that learns a hash function for each data source. Then, the patient similarity is calculated by hash codes from data sources. To avoid the potential security risk because of the attack from malicious users, we also adopt homomorphic encryption [31] to support secure patient similarity search in a federated setting. Finally, the proposed algorithm is applied and validated on real data.

## Methods

### Feature Construction

**Figure 1:**
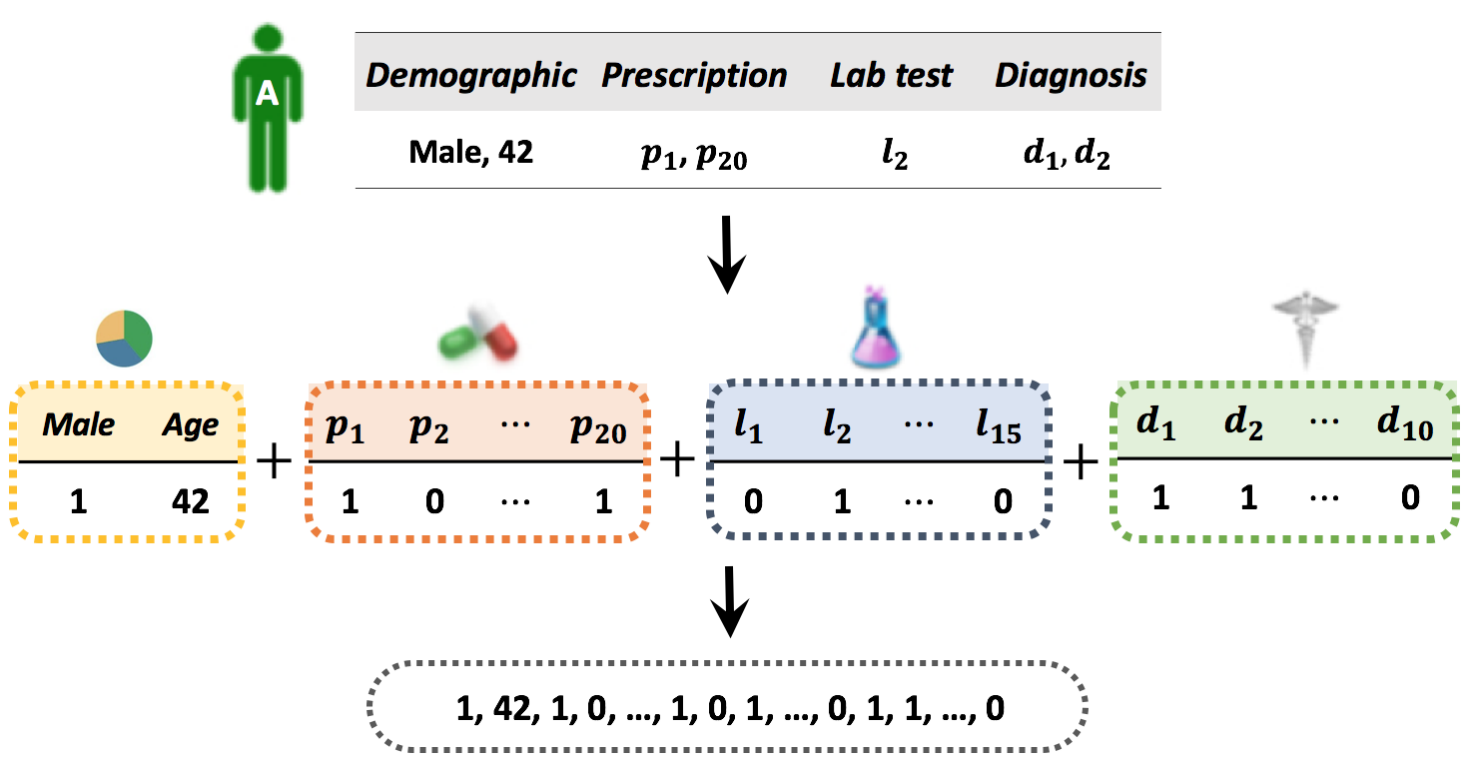
Example of feature construction. Prescription, lab test, and diagnosis are denoted by p, l, and d, respectively.

For *K* feature domains, we assume a vector-based representation for patients in every feature domain (1≤*k*≤*K*). There are different ways to construct the feature vectors: (1) for nominal features with standard dictionaries, such as diagnosis and procedure codes, we can use either binary value for presence, or code frequency within the observation period (where the features are extracted from); (2) for continuous features such as age or lab test values, we can use them as they are or we can first quantize them and treat each quantized region as a nominal feature. For example, the values of a specific lab test can be quantized as critical low, low, normal, high, and critical high; and (3) for time-evolving features, if we want to consider the temporal trends in the feature construction process, we can first construct a temporal pattern dictionary with either data-driven method or expertise knowledge, and then treat each pattern as a nominal feature. For example, if there are four types of features including two demographics, 20 prescriptions, 15 lab tests, and 10 diagnoses, we can construct a vector-based representation for patient A as shown in Figure 1. We represent gender as a binary value and age as it is. For diagnosis, prescription, and lab test, we add a one-hot representation of each event (ie, {0,1}^|C^^|^ with the number of codes | *C* |).

### Hashing

**Figure 2:**
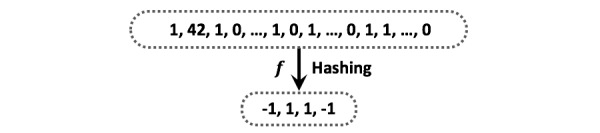
Example of hashing.

In general, hashing is an approach of transforming the data item to a low-dimensional representation, or equivalently a short code consisting of a sequence of bits (Figure 2).

Hashing technologies can be applied in many applications such as Bloom filter [32] and cryptography [33]. Similarity-based hashing [34] is one specific type of hashing that aims to preserve the data similarities in their original space with hash codes. On the basis of the availability of supervision information, a similarity-based hashing method can be categorized as unsupervised [35-40], semi-supervised [41-43], or supervised hashing [44,45]. Unsupervised methods learn hash functions purely based on data distributions. Supervised methods exploit the labeled pairwise relationship between entities to capture the high-level data semantics. Semi-supervised methods lie in between them, that is, they explore both data distribution characteristics and labeled pairwise data relationships to learn the hash functions. Most of these existing methods assume a single vector-based representation for every data object.

However, one challenge in our scenario is that the patient features are highly heterogeneous, that is, the features for characterizing the patients are of different types. In this case, it may not be effective to represent each patient as a single vector (simple concatenation will not work as different features are of different types and have different value range). There are some existing multi-modal hashing methods [46-52] that aim to derive a unified single-hash table for encoding the data objects with heterogeneous features. The problem with single-hash (or uni-hash) table is that it is difficult to discover the latent similarity components [53] derived from different feature types, which is crucial in our scenario. For example, it is important to know how similar two patients are, but also why (eg, patients A and B are similar to each other mainly because of their similar demographics and patients B and C are similar because of their similar diagnosis history and lab test values).

### Federated Patient Hashing Framework

Symbols used in this paper are listed in Textbox 1.

**Figure 3:**
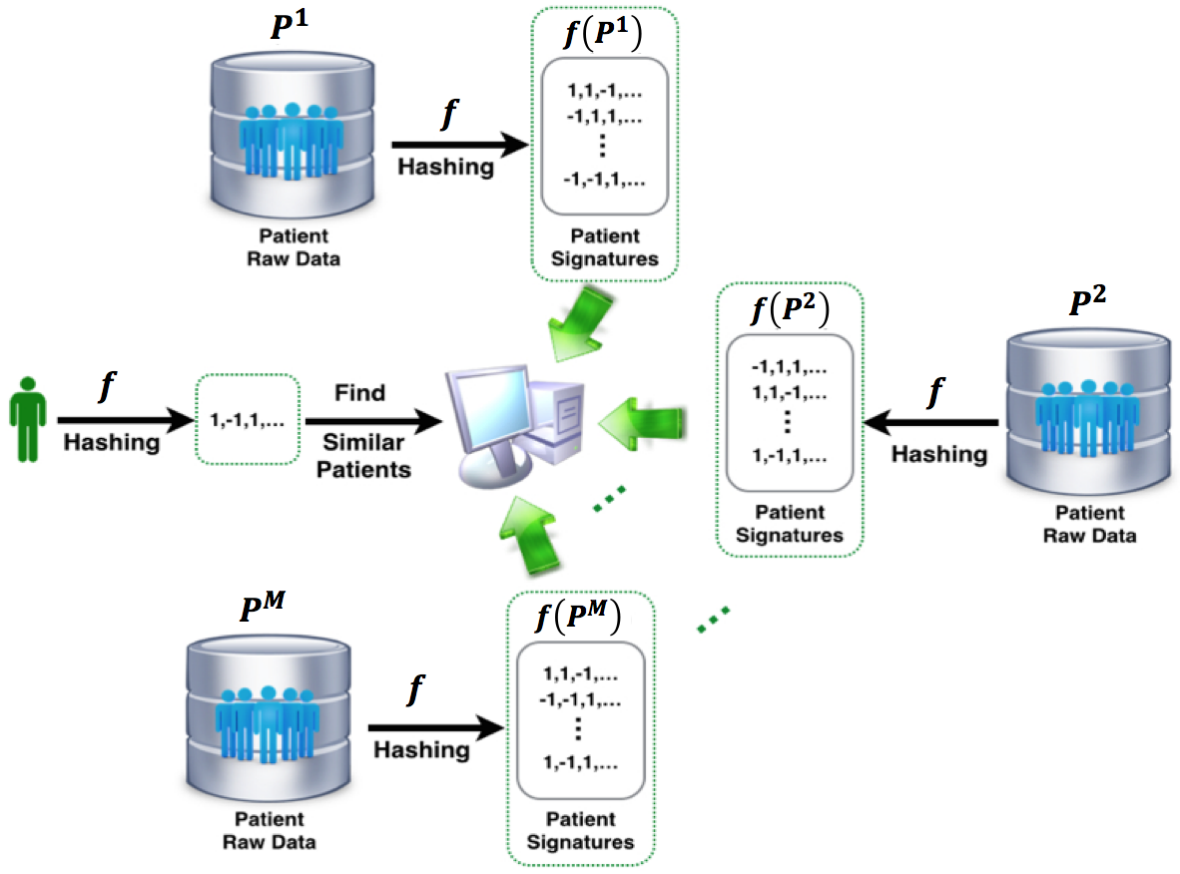
The whole process of patient matching in a federated environment. The user sends a patient matching request to the service center, which is delegated to patient data resources from several clinical sites. Due to the privacy concerns, the center does not have access to the raw patient data. All patients within different sites need to be first hashed, and the center only has the patient’s signatures after hashing. The hash functions are shared across different sites.

Figure 3 illustrates the overall federated patient matching framework. Suppose there are *M* sites with the *i*-th site *S*^i^ which owns a patient population *P*^i^. We use *p*^i^_j_ to represent the *j*-th patient in *P*^i^. Then, our problem is, given a query patient, how to retrieve similar patients from those *M* sites without explicitly accessing the patient feature vectors. Our plan is to resolve this problem using similarity based hashing, which transforms the patient’s raw features into a binary vector representing patient characteristics (patient representation learning). The pairwise patient similarities will be evaluated as the pairwise distance based on those signatures (patient similarity search). In this paper, we will focus on feature-based hashing, that is, those binary patient signatures are obtained by proper transformation from patient features. Therefore, to perform hashing, we need to first construct feature-based representation for patients.

**Figure 4:**
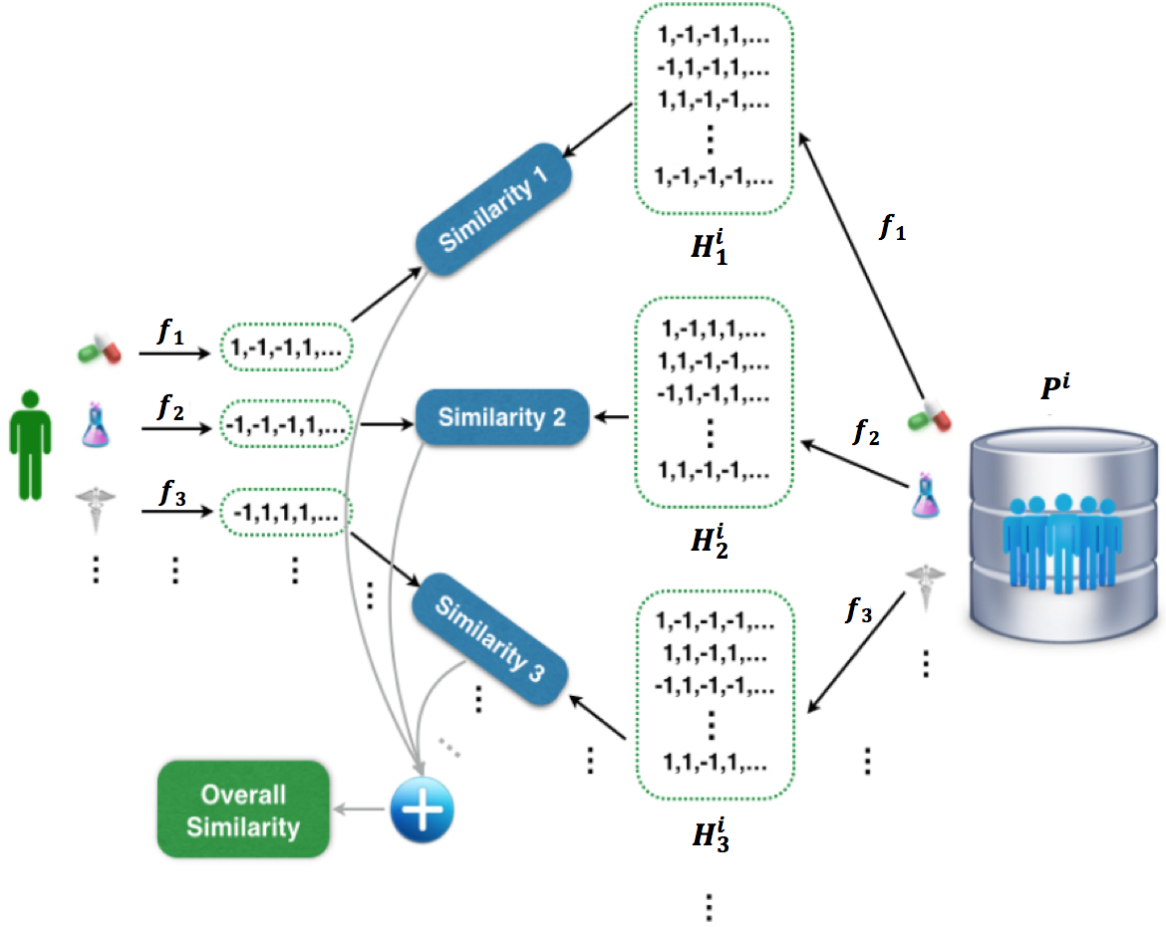
The process of calculating patient similarity with a multi-hash approach.

Without the loss of generality, we assume there are *K* different feature types to characterize every *p*^i^_j_, and we use *p*^i^_jk_ (*k*=1,2,…, *K*) to represent the *k*-th type of feature vector of *p*^i^_j_. The goal is to derive an effective computational framework for patient matching in a federated environment, and the key idea is to learn a good hash function that can transform the patient features into binary hash codes. A uni-hash table approach shown in Figures 2 and 3 is to learn only one hash function for the feature vector *f*: *R*^d^→{-1,+1}^b^, where *d* is the dimensionality of the whole feature vector, and *b* is the number of bits of the hash codes learned *d* by *f*. In this paper, we propose a multi-hash approach for patient hashing that aims to learn a hash function *f*_k_: *R*^d^_k_→{-1,+1}^b^_k_ for every patient feature type *k* (*k*=1,2,…, *K*); *d*_k_ is the dimensionality of the *k*-th feature type, and *b*_k_ is the number of bits of the learned hash codes for the *k*-th feature type. Each *f*_k_ (*k*=1,2,…, *K*) is shared across all the *M* sites. We use the sign function to construct the hash codes, that is, sign (*Q*^i^_k_)∈{-1,+1}^b^_k_^ⅹN^_i_ , where *Q*^i^_k_ is transformed numerical data from original data of *i*-th site for *k*-th type of feature *P*^i^_k_∈ *R*^d^_k_^ⅹN^_i_ by a hash function *f*_k_ that incorporates function coefficients for the *k*-th feature type *W*_k_∈^d^_k_^ⅹb^_k_; *N*_i_ is the population size of *i*-th site. How these components are formulated is described in the next paragraph in detail. We use *H*_k_^i^=sign(*Q*^i^_k_) to denote the hash codes of *k*-th feature type for the patients at *i*-th site. Figure 4 shows the process of patient similarity calculation with a multi-hash approach.

The *u*-th column of *H*_k_^i^, *h*^i^_uk_∈{-1,+1}^b^_k_ is the hash codes of *p*^i^_uk_. Then, the similarity between *p*^i^_uk_ and *p*^i^_uk_ can be evaluated as the inner product of *h*^i^_uk_ and *h*^i^_uk_ as shown in equation 1:


**(1) **
*s*
^i^_kuv_ = 1/
*b*_k_(*h*^i^_uk_)
^T^(*h*
^i^_vk_)

Thus, the overall similarity can be computed as the average of *K* similarities, as shown in equation 2, which is bounded on the interval of (−1,1).


**(2) **
*s*
^i^_uv_ = 1/
*K*∑_k_(*h*
^i^_uk_)
^T^(*h*
^i^_vk_)

Here, we suggest a general framework for learning {*W*_k_}^K^_k=1_, which is the most important component. The framework basically constructs an objective function in terms of {*W*_k_}^K^_k=1_ such as shown in equation 3, where *λ*_S_*, λ*_U_*,* and *λ*_W_ are regularizers of *S* ({*W*_k_}^K^_k=1_), *U* ({*W*_k_}^K^_k=1_), and Ω({*W*_k_}^K^_k=1_), respectively, and then minimizes (or maximizes) it:


**(3) **
*J* ({*W*_k_}^K^_k=1_) =
*Ψ* ({*W*_k_}^K^_k=1_) +
*λ*_S_
*S* ({*W*_k_}^K^_k = 1_) +
*λ*_U_
*U* ({*W*_k_}^K^_k = 1_) +
*λ*_W_Ω ({*W*_k_}^K^_k = 1_)

*Ψ* ({*W*_k_}^K^_k=1_) is a reconfiguration error term between the low-dimensional representation of the original data and hash codes, which is the main term of the objective function, and generates the hash codes from the original data, as shown in equation 4, where ||·||_F_ is a Frobenius norm [54]. On the basis of this term, the hash function in our framework is formed as *f*_k_(*P*_k_^i^)*=W*_k_^T^*P*_k_^i^*,* and this transformation results in *H*_k_^i^=sign(*Q*^i^_k_).


**(4) **
*Ψ* ({*W*_k_}^K^_k=1_) = ∑_i_∑_k_||*W*_k_^T^
*P*_k_^i^ -
*H*_k_^i^||^2^_F_

The objective function can incorporate regularizers, as well as the main term to obtain better solutions of {*W*_k_}^K^_k=1_ by (1) introducing additional information to improve either unsupervised or supervised learning if desired, (2) solving an ill-posed problem, and 3) preventing overfitting. Possible regularizers are listed as follows:

*S*({*W*_k_}^K^_k=1_) is a supervised loss term that measures the quantization loss during the hashing process when supervision information is available for the patients. Here, the supervision information could be the labels of the patients, such as the disease the patients have. For example, if both *p*^i^_u_ and *p*^i^_v_ have the same disease, then their relationship *r*^i^_uv_=1, otherwise *r*^i^_uv_=-1. Then, we can set *S*({*W*_k_}^K^_k=1_) as shown in equation 5:


**(5) **
*S* ({*W*_k_}^K^_k=1_) = ∑_i_∑_k_∑_u,v_ -
*s*
^i^_kuv_
*r*
^i^_uv_

**List of symbols.**

*M*: the number of local sites

*K*: the number of feature types (domains)

*S*^i^: *i*-th local site

*P*^i^: patient population in *S*^i^

*N*^i^: patient population size of *S*^i^

*P*^i^_k_: patient population for *k*-th type of feature in *S*^i^

*p*^i^_j_: *j*-th column of *P*^i^, *j*-th patient in *P*^i^

*p*^i^_jk_: *j*-th column of *P*^i^_k_, *k*-th type of feature vector for  *p*^i^_j_

*f*_k_: *k*-th hash function

*d*_k_: dimensionality of the *k*-th feature type

*b*_k_: the number of bits of the learned hash codes for the *k*-th feature type

*W*_k_: function coefficients of the hash function for the *k*-th feature type

*w*_ik_: *i*-th column of *W*_k_

*Q*^i^_k_: numerical data transformed from *P*^i^_k_

sign(*Q*^i^_k_): signed *Q*^i^_k_

*H*^i^_k_: hash codes for *P*^i^_k_(=sign(*Q*^i^_k_))

*h*^i^_jk_: *j*-th column of *H*^i^_k_, the hash codes of *p*^i^_jk_

Ψ({*W*_k_}^K^_k=1_): reconfiguration error term for {*W*_k_}^K^_k=1_

*S* ({*W*_k_}^K^_k=1_): supervised loss term for {*W*_k_}^K^_k=1_

*U* ({*W*_k_}^K^_k=1_): unsupervised loss term for {*W*_k_}^K^_k=1_

*Ω* ({*W*_k_}^K^_k=1_): term related to {*W*_k_}^K^_k=1_ itself

*L* (*x*, *y*): loss function between *x* and *y*

*λ*_S_: regularizer of *S* ({*W*_k_}^K^_k=1_)

*λ*_U_: regularizer of *U* ({*W*_k_}^K^_k=1_)

*λ*_W_: regularizer of *Ω* ({*W*_k_}^K^_k=1_)

*λ*: regularizer of a supervised loss term

*η*: regularizer of a Frobenius norm for *Q*

*σ*^i^_kuv_: similarity between *p*^i^_uk_ and *p*^i^_vk_

*R*^i^: pairwise relationship of *R*^i^ for labeled information

*S*^i^_k_: pairwise similarity of *P*^i^_k_

*r*^i^_uv_: relationship between *p*^i^_uk_ and *p*^i^_vk_ for labeled information

*s*^i^_kuv_: similarity between *p*^i^_uk_ and *p*^i^_vk_

*S*_L_*(Q*^i^_k_*)*: approximated sign function for *Q*^i^_k_

The possible choices of supervised loss term could be any loss function *L* (*x,y*), and examples include *L(x,y)=-xy* and well-known binary loss functions such as (1) logistic loss, *L* (*x,y*)=log(1+exp(- *xy*)) and (2) hinge loss, *L* (*x,y*)=max(0,1-*xy*).

Note that *U*({*W*_k_}^K^_k=1_) is an unsupervised term that exploits the intrinsic data distribution and enforces the resultant hash codes to comply with the distribution. For example, we can request similar patients to have similar hash codes on each feature type. This can be achieved by minimizing the below regularizer, as shown in equation 6, where *σ*^i^_kuv_ is a similarity between *p*^i^_uk_ and *p*^i^_vk_ based on, for example, a Gaussian function for continuous valued features or a cosine function after Term Frequency-Inverse Document Frequency normalization on bag-of-code (eg, diagnosis code or procedure code):


**(6) **
*U* ({*W*_k_}^K^_k=1_) = ∑_i_∑_k_∑_u,v_
*σ*
^i^_kuv_||*h*
^i^_uk_-
*h*
^i^_uk_||^2^_F_

Ω({*W*_k_}^K^_k=1_) is a term related to {*W*_k_}^K^_k=1_ themselves, which is independent of the patient features. Examples of Ω({*W*_k_}^K^_k=1_) include (1) Frobenius norm regularizer ∑^K^_k=1_||*W*_k_||^2^_F_, which can be used for improving the numerical stability of the solution process and (2) orthogonality regularizer ∑^K^_k=1_∑_i≠j_||*w*^T^_ik_*w*_jk_||^2^, where *w*_ik_ is the *i*-th column of *W*_k_, which can encourage the diversity of the learned hash codes and thus improve their representation effectiveness.

**Figure 5:**
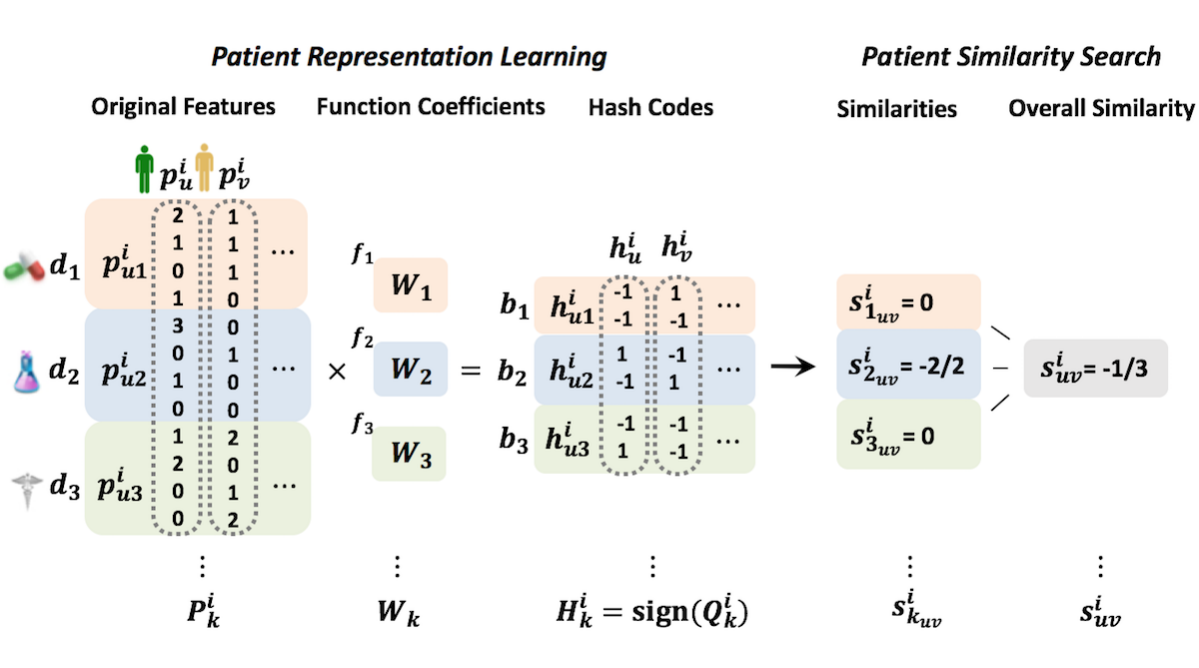
Example of transformation of patient vectors into hash codes and computation of similarity between hash codes.

Figure 5 shows a running example of the proposed hashing methodology. Such optimization problems can be solved with Block Coordinate Descent technologies [55], with {*W*_k_}^K^_k=1_ as variable blocks that alternatively update *W*_k_ (1≤*k*≤*K*) one by one. Moreover, as different sites are continuously receiving new patients (or new patient features), we will need to continuously update the hash functions as well. Fortunately, as can be observed from equations 4, 5, and 6, those terms are fully decomposable with respect to different sites. Therefore, we can update the hash functions in an asynchronous manner, that is, we can update the current {*W*_k_}^K^_k=1_ as soon as new patient data is received on site *i*.

### Privacy-Preserving Patient Representation Learning in a Federated Setting

Without loss of generality, let us instantiate the objective function with the regularizer *λ* of empirical error on the labeled data for a family of hash codes; this choice might be the most basic approach to similar patient learning based on the fact that supervised learning is more commonly used than unsupervised learning because data generated in the medical field usually have label information.

When solving the initiated objective function, two possible problems because of the sign function for *Q* arise. First, *Q* may not be a unique solution, and thus, the objective function is difficult to converge without considering any regularizer about *Q*. We add a Frobenius norm regularizer *η* to solve this problem. In addition, the objective function *f(W,Q)* is nondifferentiable in terms of *Q*. We can approximate the sign function with the surrogate function. Then, we have the final objective function, as shown in equation 7, where *R*^i^∈ *R*^N^_i_^ⅹN^_i_ is the pairwise relationship of *P*^i^ for labeled information:


**(7) **
*f* (*W*,
*Q*) = min∑_i_∑_k_||*W*_k_^T^
*P*
^i^_k_
*-S*_L_(
*Q*
^i^_k_)||^2^_F_ +
*λ* ∑_i_∑_k_ tr(*-S*_L_(
*Q*
^i^_k_)
*R*
^i^
*S*_L_(
*Q*
^i^_k_)^T^
*) + η* ∑
_i_∑
_k_||
*Q*
^i^_k_||^2^_F_

If both *p*^i^_u_ and *p*^i^_v_ have the same disease, then their relationship *r*^i^_uv_=1, otherwise *r*^i^_uv_=-1, and *S*_L_(·) is the surrogate function, as shown in equation 8, where ∘ is the hadamard (elementwise) product:


**(8) **
*S*_L_ (*Q*
^i^_k_) = (*Q*
^i^_k_∘
*Q*
^i^_k_
* + ξ*)
^-1/2^∘*Q*
^i^_k_

The detailed process to derive the final objective function is given in Multimedia Appendix 1 (Note ⊗ is the Kronecker product [56]). The objective function for {*W*_k_}^K^_k=1_ and {*Q*^i^_k_}^K,M^_k,i=1_ can be solved one by one iteratively as variable blocks [55] by using the Newton-Raphson method [57] until the estimates converge. To be specific, this approach first allows us to update *W*_k_ for each of *k* (*k*=1,2,…, *K*) with other *W*_l_ for all *l* (1≤*l≠k*≤*K*) and *Q* being fixed:


**(9) **
*W*
^new^_k_ =
*W*_k_ - (∂
^2^
*f* /∂
*W*
^2^_k_)
^-1^∂
*f* /∂
*W*_k_

Then, similarly, we update *Q*^i^_k_ for each combination of (*i*, *k*) (1≤*i*≤*M*,1≤*k*≤*K*) with other combinations of (*j*, *l*) (1≤*j*≠*i*≤*M*,1≤*l*≠*k*≤*K*) and *W* being fixed:


**(10) **
*Q*
^i,new^_k_ =
*Q*
^i^_k_ - (∂
^2^
*f* /∂
*Q*
^i^_k_^2^)
^-1^∂
*f* /∂
*Q*
^i^_k_

The derivation process for the first and second derivatives of *W* and *Q* is described in Multimedia Appendix 1. As derivatives are linearly decomposable by sites *i*, the objective function defined in equation 7 can be computed in a distributed manner. This means the optimization only requires locally computed statistics to be delivered to estimate the {*W*_k_}^K^_k=1_ iteratively until convergence.

The time complexity at each iteration depends on feature type *k* and site *i*. When updating *W*_k_ for each of *k* (1≤k≤*K*) with other *W*_l_ for all *l* (1≤*l*≠*k*≤*K*) and *Q* being fixed, the time complexity is *O* (*d*_k_^3^) because each site has to inverse the *d*_k_ⅹ *d*_k_ Hessian matrix. When updating *Q*^i^_k_ for each combination of (*i*, *k*) (1≤*i*≤*M*, 1≤*k*≤*K*) with all other combinations of (*j*, *l*) (1≤*j*≠*i*≤*M*, 1≤*l*≠*k*≤*K*) and *W* being fixed, the time complexity is *O* (*b*_k_^3^*N*_i_^3^) because *S*^i^ has to inverse the *b*_k_*N*_i_ⅹ*b*_k_*N*_i_ Hessian matrix. Therefore, parameters that have a significant effect on time complexity include original and projection dimensions by feature type and population size by site. Other parameters such as the number of sites *M* and the number of feature types *K* along with the number of iterations are excluded in the big *O* notation because they are just constants. That is unless the number of site or the number of feature type goes to infinity, it only has a small impact on the complexity.

### Privacy-Preserving Patient Similarity Search in a Federated Setting

**Figure 6:**
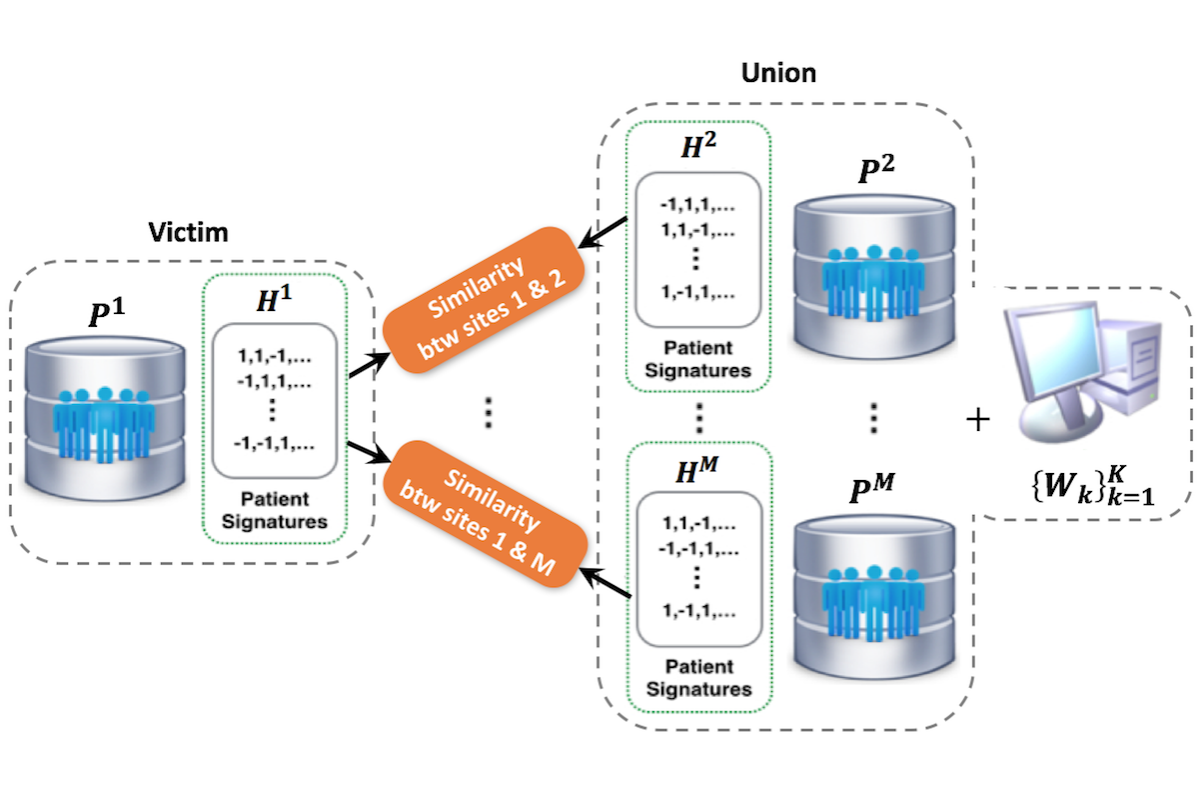
Example of potential privacy leakage in patient similarity search across sites.

To find similar patients across sites, hash codes for each site *H*^i^ (ie, {*H*^i^_k_}^K^_k=1_ have to be exchanged across institutions originally. However, when all other sites expect for *i*-th site receive *H*^i^ for similarity search, the patient-level information of *i*-th site can be leaked by equation 4; other sites and a server can be united for reverse engineering to extract *P*^i^because they have both {*W*_k_}^K^_k=1_ and *H*^i^, as well as their information in equation 4. Figure 6 illustrates the situation mentioned.

**Figure 7:**
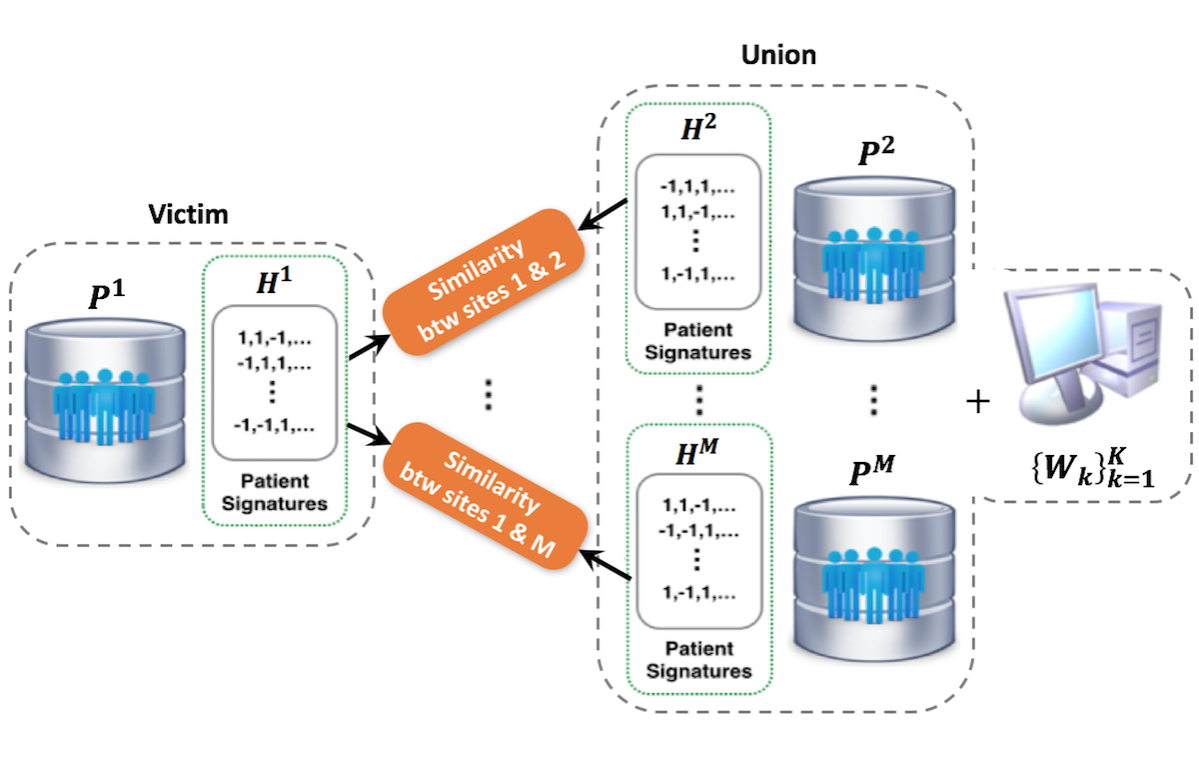
Privacy-preserving patient similarity search by homomorphic encryption; green key: encryption (public) key, blue key: decryption (private) key.

Therefore, we suggest the way to search similarity among different sites by avoiding revealing *H*^i^_k_ but able to compute similarities based on *H*^i^_k_. We introduce homomorphic encryption specifically that is a form of encryption where a specific algebraic operation performed on the plaintext is equivalent to another algebraic operation performed on the cipher-text, and an encrypted result, when decrypted, matches the result of the same operation performed on the plaintext. Unlike traditional encryption schemes that do not allow any computations to be performed on the cipher-text without first decrypting it, homomorphic encryption allows computations to be performed without decrypting the data. The results of the computations remain encrypted and can only be read and interpreted by someone with access to the decryption key. Therefore, it is appropriate to use homomorphic encryption in our case that other sites and a server can attack maliciously. It enables cross-site comparison of health care statistics with protecting privacy for each site. The procedure of homomorphic encryption in this paper is summarized as follows: first, *i*-th site encrypts hash codes for its query data and delivers encrypted codes to *j*-th site. Next, *j*-th site performs the computation between delivered encrypted codes of *i*-th site and encrypted codes of *j*-th site without a decryption key and sends the computed value to *i*-th site. Finally, *i*-th site decrypts the value to get the hamming distance of hash codes between query data and data of *j*-th site. Each site is restricted to only answer the hamming distance to avoid the risk of privacy leakage. This process is depicted in Figure 7.

We note that homomorphic encryption provides an extra layer of privacy protection especially during patient similarity search.

### Security

There are several participants in our framework.

- Data custodians (DCs) represent institutions or hospitals who have access to patient data and would like to collaborate in learning about similar patients.
- Crypto service provider (CSP) generates public and private keys. The public key is provided to the data custodians to safeguard the intermediary statistics.
- Cloud server (CS) computes over summary statistics from individual data custodians to obtain a global patient similarity model.

Our goal is that a DC does not learn patient-level information from other DCs during the process. We also want to ensure CS cannot infer patient-level information from the data. We assume a CSP is trustworthy and provides encryption keys (public and private). In the threat model, we assume the CS to be semi-honest, that is, it is honest to follow the protocol but curious about patient’s private information while executing the protocol. We make the following basic assumptions: (1) DC and CS do not collude, (2) CS and CSP also do not collude, and (3) DC always receives correct keys from the CSP. To evaluate the security of our system, it is assumed that the security of the system is compromised if patient-level data or intermediary statistics that can infer patient-level data are leaked. CSP is only involved in generating public and private keys and transferring those keys to DCs, and no access to unintended fine-grained local information is involved in this process.

The leakage is related to computation of {*W*_k_}^K^_k=1_, and possible scenarios according to the participants are as follows:

- Leakage to CSP in each computation: CSP does not participate in computation at all. Therefore, there is no leakage.
- Leakage to DC in each computation: each DC cannot indirectly learn patient data from other DCs only with {*W*_k_}^K^_k=1_ and its local information {*W*_k_}^K^_k=1_ and {*Q*^i^_k_}^K^_k=1_. If all DCs except for one collude, it is infeasible for the other DCs to reconstruct *P*^i^_k_ of that one DC because the first and second derivatives of *W*_k_ have a nonlinear relationship for *P*^i^_k_. Specifically, it is not possible to specify a certain matrix only given information of covariance matrix because of insufficient equations. They also do not have information (first and second derivatives) about *Q*^i^_k_.
- Leakage to CS in each computation: CS cannot infer patient data from {*W*_k_}^K^_k=1_. Even though CS receives local information for the first and second derivatives of {*W*_k_}^K^_k=1_, it is infeasible for CS to recover {*P*^i^_k_}^K^_k=1_ for the same reason as the collusion among DCs. In finding similar patients, hash codes for each site {*H*^i^_k_}^K^_k=1_ have to be exchanged across institutions originally, but the use of homomorphic encryption prevents direct exchange of hash codes {*H*^i^_k_}^K^_k=1_ between DCs, and thus, there is no leakage.

## Results

### Experimental Setting

We conducted experiments to validate our proposed method on real data. For comparison, we assumed two different systems against our system according to connection among *M* sites: open and closed system. In the open system, *M* sites can exchange their patient’s information without any restrictions; in the closed system, each site can only utilize patient's information in each site. Ours is in the middle of two systems. For better understanding of these systems, let us assume that there are three sites A, B, and C with the same number of patients *N*. In this situation, an open system means that every site can access the complete information of the entire patient cohort (3ⅹ *N*), including information from other sites as well, and thus, three sites work like one site without any concerns on privacy. On the other hand, closed system indicates that each site can only access its patient-level information (*N*) exclusively. Open system and closed system are derived based on an idealistic situation and a realistic situation, respectively, and our system is in between these two systems, which cannot access patient’s information from other sites but can utilize it through { *W*_k_}^K^_k=1_. Then, we predicted the incidence of a certain disease and compared the standard *κ*-nearest neighbor (*κ*-NN) classification results based on hamming distance of multi-hash codes from our system with those based on hamming distance of multi-hash codes from open and closed systems, as well as uni-hash codes from open and closed systems. We also provided baseline results based on four similarity distances of raw data without using hashing for open and closed systems: Euclidean, cityblock, cosine, and correlation. We utilized five-fold cross validation (CV) that randomly splits patients into five folds with the equal size; we used four folds for training, and one fold for testing. As an evaluation measure, we used the area under the curve (AUC) where the true positive rate (TPR; ie, the number of true positives divided by the sum of true positives and false negatives) is plotted against the false positive rate (ie, the number of false positives divided by the sum of false positives and true positives) at various thresholds. AUC as a summarized single value for the curve has desirable properties that are independent to the threshold and invariant to a priori class probability distributions. An area of 1 represents a perfect model, and an area of 0.5 represents a worthless model. As we repeated CV ten times, we obtained ten vectors consisting of probabilities based on *κ* nearest neighbors’ voting. The program was implemented by MATLAB 2015b (MathWorks).

### Temporal Sequence Construction

**Figure 8:**
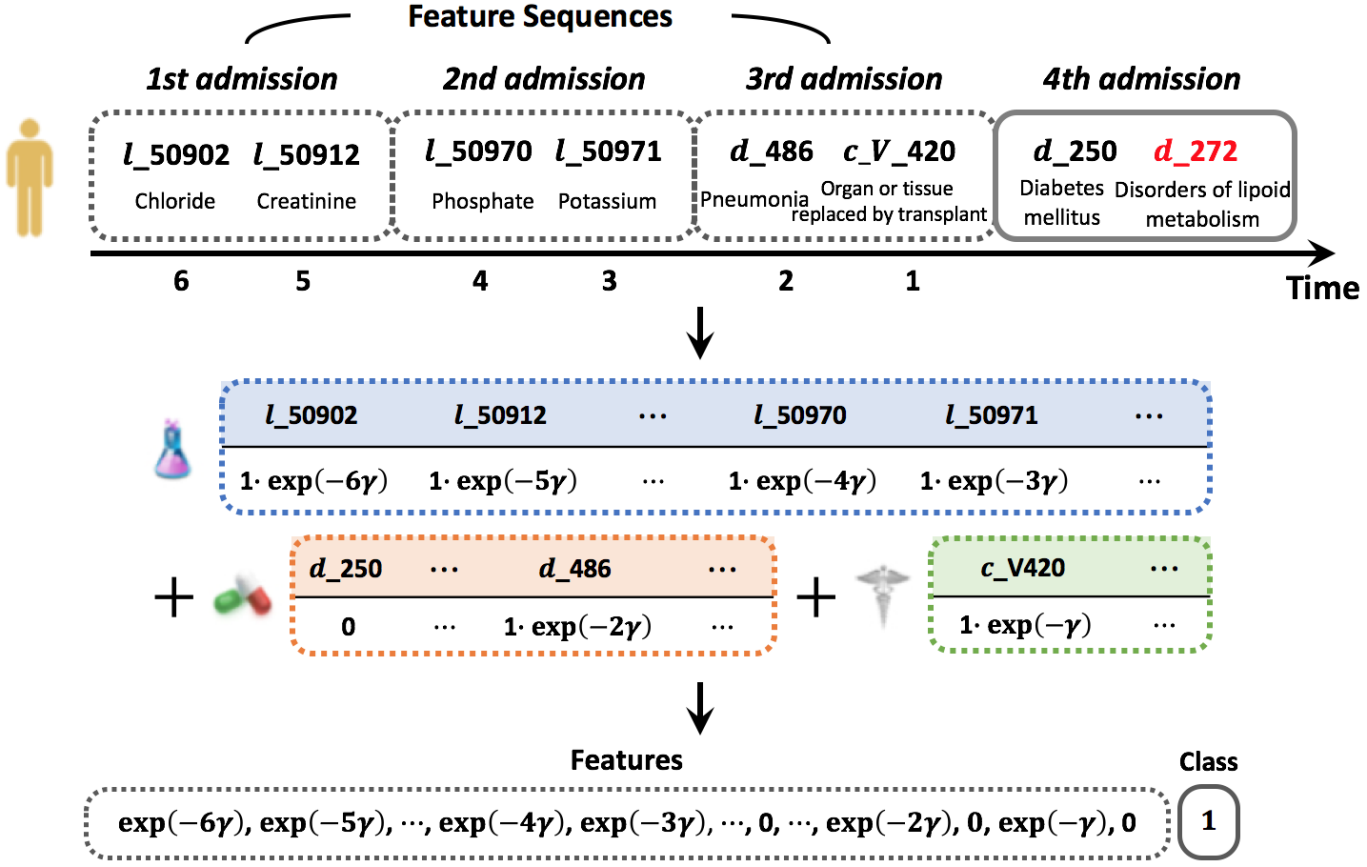
Example of constructing temporal sequence with target disease in red and its vector representation.

A sequence is composed of lab tests, prescriptions, diagnoses, conditions, and symptoms that were given to a patient in multiple hospital admissions. We only extracted common lab tests, prescriptions, diagnoses, conditions, and symptoms (prefixed with “ *l*_,” “ *p*_,” “ *d*_,” “ *c*_,” and “ *s*_,” respectively). We used the International Classification of Diseases, 9th revision (ICD-9) level 3 codes instead of level 4 or 5 to avoid extreme sparsity of diagnoses. We assumed space in time between all events to be same. Then, we constructed data for incidence of a target disease as follows: for patients in which a target disease occurs, we sliced the very admission that includes the diagnosis event of a target disease out of the sequence, and used only events before that admission as a feature sequence. For other patients, we used all events. We utilized temporal information of a sequence to make a time-decayed vector representation; when we add a one-hot representation for each event, it is multiplied by the time decaying function (ie, exp(-*γt*) with the decay constant *γ*) that enables to weaken the effect of old event but to strengthen the effect of recent event. A graphical illustration of this sequence and its vector representation is presented in Figure 8.

### Multiparameter Intelligent Monitoring in Intensive Care-III Database

We used Multiparameter Intelligent Monitoring in Intensive Care-III (MIMIC-III) database that contains health-related data associated with 46,520 patients and 58,976 admissions to the intensive care unit of Beth Israel Deaconess Medical Center from 2001 to 2012. The database consists of detailed information about patients, including demographics such as gender, age, and race; admissions; lab test results; prescription records; procedures; and discharge ICD diagnoses. On the basis of this database, we randomly selected several common diseases (ie, diseases with relatively large number of positives) as a target disease to verify that our method can perform well in general not only for a specific disease. Then, we extracted temporal sequences and constructed following six feature vectors (*K*=6) for patients in *i*-th site: demographic information *P*^i^_1_∈*R*^d^_1_^ⅹN^_i_, lab results *P*^i^_2_∈ *R*^d^_2_^ⅹN^_i_, diagnoses *P*^i^_3_∈*R*^d^_3_^ⅹN^_i_, prescriptions *P*^i^_4_∈*R*^d^_4_^ⅹN^_i_, conditions *P*^i^_5_∈*R*^d^_5_^ⅹN^_i_, and symptoms *P*^i^_6_∈ *R*^d^_6_^ⅹN^_i_. Time decay constant *γ* was set to 0.01. We note that the feature vector of diagnoses in each dataset does not include its outcome of interest. Information of original datasets is described in Table 1.

To test three-site scenario, we made datasets balanced and horizontally partitioned the dataset into three, assuming data are evenly partitioned among sites (*M*=3), *P*^1^_k_∈*R*^d^_k_^ⅹ125^, *P*^2^_k_∈*R*^d^_k_^ⅹ125^, and *P*^3^_k_∈*R*^d^_k_^ⅹ125^ for every *k*=1,…6; federated system is needed when each institution has a limited sample size that is not enough for an analysis. In addition, from the complexity analysis, time to implement the algorithm exponentially increases in proportion to the number of patients. On the basis of these, we randomly selected and placed 125 patients in each site. Then, we predicted the incidence of five diseases independently. We set parameters for regularizers *λ*=0.5 and *η*=10^-3^ in common. In addition, for multi-hash approach, we reduced the original dimensions for each feature to ten (ie, *b*_k_=10 for *k*=2,…,6) except for the demographic feature that was reduced to two (ie, *b*_1_=2), and for uni-hash approach we reduced the total dimensionality to the sum of projection dimensions in multi-hash approach (ie, *b*=52). We note that the results would be robust to the projection dimensionality unless we have too many or too few of it. Table 2 shows the results of *κ*-NN with *κ*=3 based on hamming distance for the following configurations: our system, open and closed systems with multi-hash, as well as open and closed systems with uni-hash.

**Table 1:** Description of five datasets from Multiparameter Intelligent Monitoring in Intensive Care-III (MIMIC-III) database.

Disease	Data size (negative or positive)	Dimension (*d*_k_, *k*=1,…,6)
Disorders of lipoid metabolism	4546/2990	(12,204,814,1338,262,170)
Hypertensive chronic kidney disease	5652/1884	(12,204,822,1338,266,169)
Cardiac dysrhythmias	3878/3658	(12,204,817,1338,263,169)
Heart failure	4167/3369	(12,204,819,1338,265,169)
Acute renal failure	4182/3354	(12,204,809,1338,268,170)

**Table 2:** Averaged area under the curve (AUC) with SD of *κ*-NN (*κ*=3) based on hamming distance from our, open and closed systems with multi-hash approach and from open and closed systems with uni-hash approach and based on cosine distance from open and closed systems.

Disease	Multi-hash			Uni-hash		Baseline	
	Our system, Averaged AUC (SD)	Open system, Averaged AUC (SD)	Closed system, Averaged AUC (SD)	Open system, Averaged AUC (SD)	Closed system, Averaged AUC (SD)	Open system, Averaged AUC (SD)	Closed system, Averaged AUC (SD)
Disorders of lipoid metabolism	0.9330 (0.0086)	0.9343 (0.0125)	0.9002 (0.0285)	0.9159 (0.0255)	0.8486 (0.0271)	0.8079 (0.0222)	0.7945 (0.0308)
Hypertensive chronic kidney disease	0.9078 (0.0346)	0.9283 (0.0432)	0.8538 (0.0421)	0.9270 (0.0350)	0.8501 (0.0305)	0.7823 (0.0261)	0.7762 (0.0262)
Cardiac dysrhythmias	0.9135 (0.0287)	0.9368 (0.0492)	0.8833 (0.0397)	0.9072 (0.0414)	0.8236 (0.0328)	0.7695 (0.0151)	0.7340 (0.0343)
Heart failure	0.9058 (0.0282)	0.9351 (0.0326)	0.8798 (0.0414)	0.9089 (0.0376)	0.8471 (0.0248)	0.7986 (0.0292)	0.7733 (0.0421)
Acute renal failure	0.9169 (0.0397)	0.9477 (0.0374)	0.8637 (0.0320)	0.8821 (0.0403)	0.7929 (0.0378)	0.7434 (0.0380)	0.7289 (0.0341)

**Figure 9:**
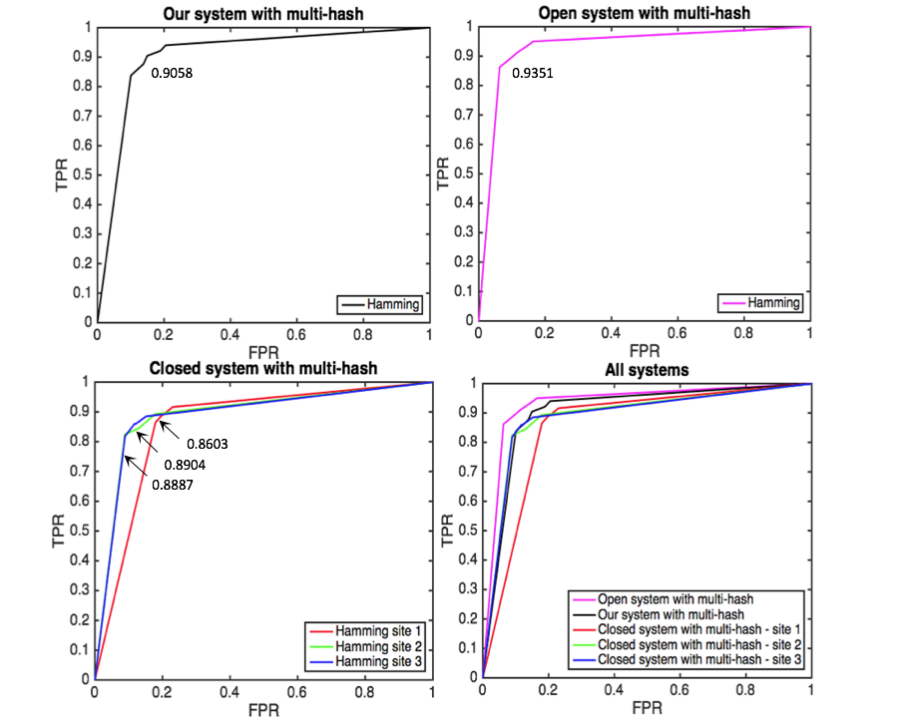
Averaged area under the curve (AUC) of κ-NN (κ=3) for heart failure based on hamming distance from our, open and closed systems with multi-hash approach.

Additionally, Table 2 presents a baseline result based on cosine distance obtained from open and closed systems, which has the highest AUC among baseline results. We note that the results for closed systems are the average of three sites.

**Figure 10:**
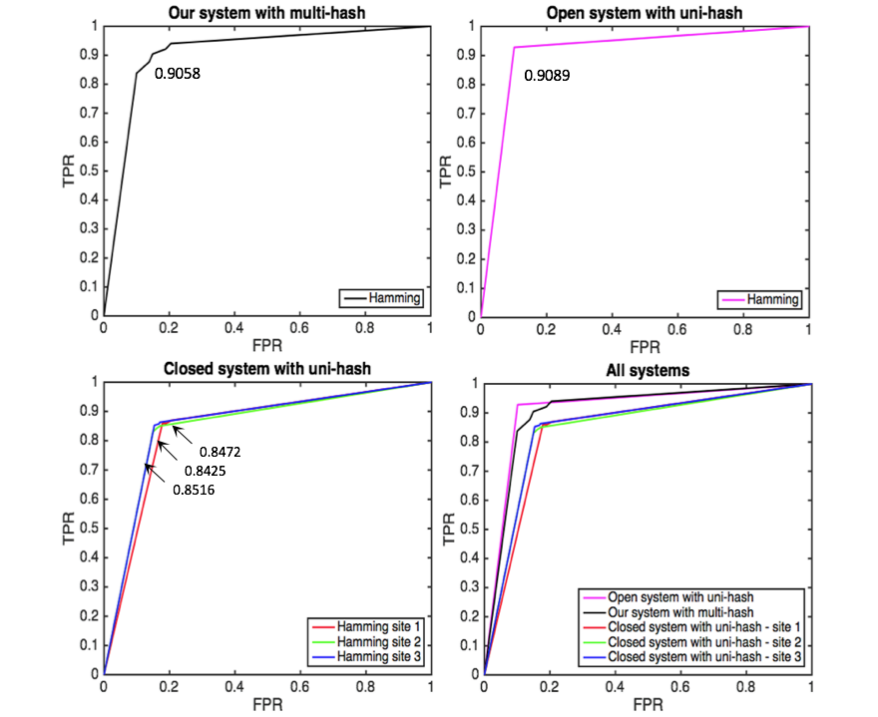
Averaged area under the curve (AUC) of κ-NN (κ=3) for heart failure based on hamming distance from our system with multi-hash approach and open and closed systems with uni-hash approach.

**Figure 11:**
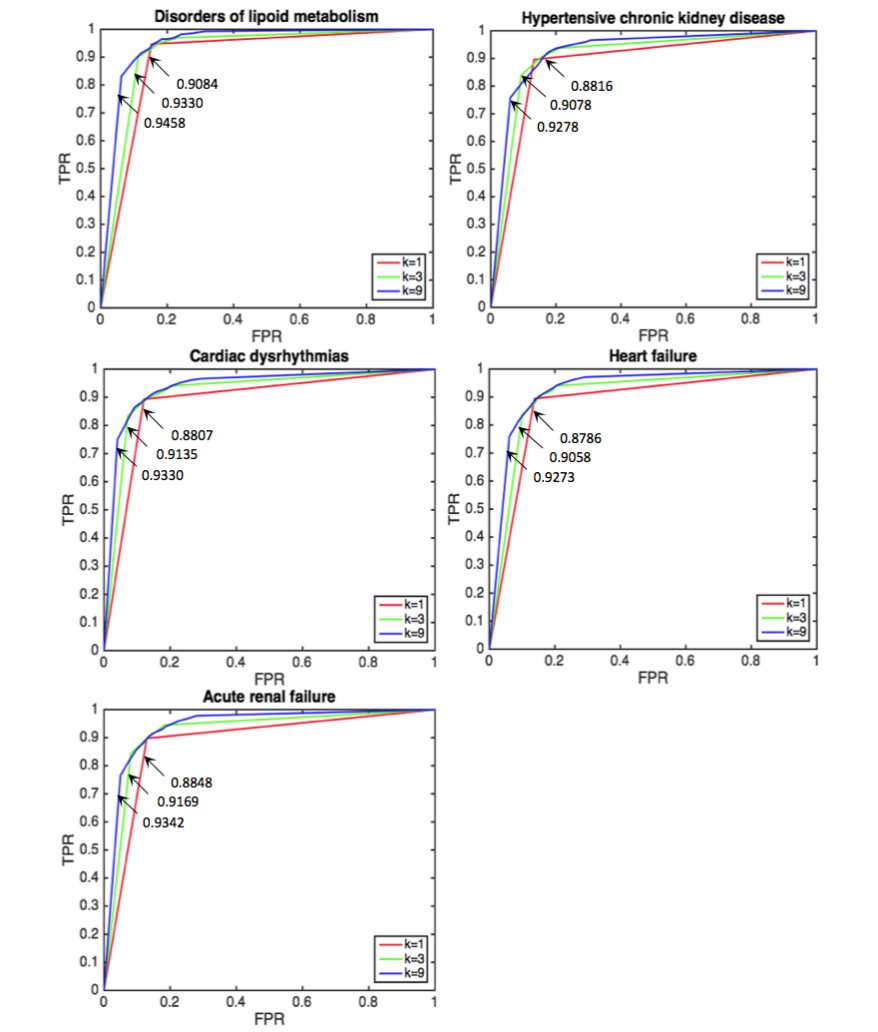
Averaged area under the curve (AUC) of κ-NN with different κ (κ=1,3,9) for five diseases from our system.

Figure 9 shows the comparison for heart failure of our open and closed systelabelms with multi-hash approach as an example. The prediction performance of our system is moderate between those of open and closed systems. It is encouraging that our system approaches open system without sharing local data. Figure 10 also shows the comparison result for heart failure of our system with multi-hash approach and open and closed system with uni-hash approach. We can see the superior performance of our system over closed system as before. However, in this case, our system is comparable with open system and even outperformed it for three diseases; this may come from multi-hash approach is more effective than uni-hash approach to construct context-specific hash codes. Figure 11 shows the results of our system with different *κ*. The detailed results with different *κ* are presented in Multimedia Appendix 2. AUC generally increases as *κ* increases.

However, in real life, different sites have a different specialty and have a different distribution in patient data. To see how our platform works in random and skewed distribution, we differentiated the ratio of samples having negative and positive classes by site. We assumed that three sites, respectively, have 10%, 30%, and 50% of positive class for five diseases. Note that all other settings including the number of sites and patients for each site, projection dimensions, and parameters were set the same as before to test only the change originated from the class imbalance and for experimental convenience; we omitted the uni-hash approach, which is expected to have the similar trend about multi-hash approach to that shown in Table 2. Table 3 shows the averaged AUC results from *κ*-NN with *κ*=3 based on hamming distance for our system, open and closed systems with multi-hash, and based on cosine distance for open and closed systems with raw data. For more elaborate comparison, F1, sensitivity (ie, TPR), and specificity (ie, the number of true negatives divided by the sum of true negatives and false positives) [58] were also measured along with AUC (Multimedia Appendix 3); F1 is the harmonic mean of precision and recall where it reaches its best value at 1 and worst at 0. It can be interpreted as weighted average of the precision (ie, the number of true positives divided by the sum of true positives and false positives) and recall (ie, TPR, sensitivity) with their equal contribution.

**Table 3:** Averaged area under the curve (AUC) with SD of *κ*-NN (*κ*=3) based on hamming distance from our, open, and closed systems with multi-hash approach and based on cosine distance from open and closed systems.

Disease	Multi-hash			Baseline	
	Our system, AUC (SD)	Open system, AUC (SD)	Closed system, AUC (SD)	Open system, AUC (SD)	Closed system, AUC (SD)
Disorders of lipoid metabolism	0.8056 (0.0386)	0.8309 (0.0412)	0.7629 (0.0295)	0.7525 (0.0212)	0.7104 (0.0187)
Hypertensive chronic kidney disease	0.7637 (0.0367)	0.7924 (0.0209)	0.7275 (0.0266)	0.7296 (0.0215)	0.7141 (0.0207)
Cardiac dysrhythmias	0.7840 (0.0301)	0.7937 (0.0228)	0.7659 (0.0223)	0.7638 (0.0198)	0.7385 (0.0188)
Heart failure	0.8287 (0.0283)	0.8832 (0.0278)	0.7459 (0.0331)	0.7735 (0.0206)	0.6778 (0.0213)
Acute renal failure	0.8239 (0.0326)	0.8704 (0.0335)	0.7558 (0.0263)	0.7304 (0.0218)	0.7415 (0.0225)

**Table 4:** Averaged execution time of each basic cryptographic operation for five diseases.

Operation	Time (seconds)				
	Disorders of lipoid metabolism	Hypertensive chronic kidney disease	Cardiac dysrhythmias	Heart failure	Acute renal failure
Homomorphic encryption	1.9	2.2	2.2	2.3	2.2
Initialization	5.2	6.3	5.8	6.5	6.0
Comparison	994.2	1243.9	1067.1	1131.7	1066.5
Homomorphic decryption	0.4	0.4	0.4	0.4	0.4

Most of the results can be interpreted in the same context as Table 2, but it should be noted that the degree of performance degradation in our system (~13%) is greater than that at baseline (~5%). Given these results from open and closed systems, as well as our system with multi-hash approach, accuracy might be lost because of the instability caused by updating weights {*W*_k_}^K^_k=1_ with information from skewed distributions. However, it is encouraging that sensitivity is obtained stably in multi-hash approach rather than baseline. Sensitivity is an important measure in medical analysis because it is much more dangerous to diagnose that the disease has not occurred even though it has already developed than the opposite case. The fact that F1 is significantly larger is consistent with this. Therefore, considering all the results, we believe that our system is a useful alternative.

Next, we performed secure data aggregation and data comparison among different sites in a federated setting by which each site is able to retrieve its hamming distance under certain criteria in a privacy-preserving manner. In our experiments with balanced data, each row has 52 bits (hash code), and a 128-bit encryption key is used for homomorphic encryption. We measured the execution time of some key cryptographic operations in a workstation with an Intel 2.5 GHz CPU, where all the results are averaged over five-fold CV of total time for six cases (three test sets by two training sets). The execution time of each basic cryptographic operation has been profiled and shown in Table 4.

We confirmed that the calculated similarities across sites are the same when exchanging raw {*H*^i^_k_}^K^_k=1_ directly with each other (ie, without homomorphic encryption) or exchanging encrypted {*H*^i^_k_}^K^_k=1_ (ie, with homomorphic encryption) with each other. Therefore, the results after homomorphic encryption were obtained exactly the same as the results in Tables 2 and 3 and Figures 9 to 11 without any privacy leakage.

## Discussion

### Principal Findings

There are several limitations in the proposed framework. When learning hash functions, the assumption is that each site has common feature events that should be needed. However, different sites, for example, hospitals, may have different event types, and additionally, the notation system for each event type cannot be standardized except for diagnoses, symptoms, and conditions that are based on ICD-9. Even though we have the limitation of common feature events, we believe that our methodology can be still useful for cooperating hospitals eager to find similar patients across sites at the point of care. We are planning to develop a new and more practical approach to relax this assumption.

Basically, our system works better when all the participants have similar distributions. However, we have confirmed through the imbalance class experiment that our system still works well with different distributions, as well at the cost of some performance degradation. We will address more generalized imbalance data problem in future work.

Next, even if we have computational benefits by adopting a multi-hash approach compared with a uni-hash approach, and the computational complexity is not prohibitive in practice, a technical challenge still remains in scalable hash function learning when the sample size and the feature dimensionality are large. This is because the complexity for inverting Hessian matrices in our algorithm is affected by the sample size and the feature dimensionality. This is an expensive operation of time complexity and requires a lot of memory. We can solve this problem by using parallelization or graphics processing units or utilizing a gradient descent method that replaces the inversion of Hessian matrix with a constant or a variable varying with the iteration number.

We demonstrated the feasibility of privacy-preserving similarity search, and the experiments were conducted on a single machine (with different processes) to serve as a proof of concept. In practice, we need to deploy the algorithm in multiple computers, and that is a trivial task. We will execute this algorithm using secure multiparty computation such as in the Secure Multi-pArty Computation Grid LOgistic REgression [59] in future work.

We have also listed several limitations to consider for more elaborate future work. When constructing temporal sequences, it assumes the sequence events are sampled at the same frequency for simplicity, which means the temporal effect has not been represented in this work. We roughly determined parameters of projection dimension and decay factor, which might not be optimal. In our experiment, we used 3-digit ICD to show a proof of concept, but the granularity of the ICD code will affect the performance in real applications, especially if the interest is related to the rare ones.

### Conclusions

We proposed a federated patient hashing framework and developed a privacy-preserving patient similarity learning algorithm. This technique allows to learn hash codes for each patient reflecting information of different sites without sharing patient-level data. Using MIMIC-III database, we conducted experiments to demonstrate the accuracy and usability of the proposed algorithm. By utilizing the multi-hash approach, our algorithm obtained more usable and practical results than the uni-hash approach. To avoid privacy leakage in patient similarity search, we also applied homomorphic encryption able to calculate the hamming distance without transmitting hash codes. As a result, we confirmed the same results without any privacy leakage.

## Appendix

Multimedia Appendix 1 Privacy-preserving patient representation learning in a federated setting.

Multimedia Appendix 2 Prediction performance of balanced class datasets.

Multimedia Appendix 3 Prediction performance of imbalanced class datasets.

Multimedia Appendix 4 Prediction performance of balanced class datasets.

## Abbreviations

AUC: area under the curve
CS: cloud server
CSP: crypto service provider
CV: cross validation
DC: data custodian
EHR: electronic health record
ICD-9: International Classification of Diseases, 9th revision
MIMIC-III: Multiparameter Intelligent Monitoring in Intensive Care-III
TPR: true positive rate
