# Supplementary material for: Privacy-Preserving Patient Similarity Learning in a Federated Environment: Development and Analysis
Source: JMIR Med Inform. 2018 Apr 13;6(2):e20. doi: 10.2196/medinform.7744 (PMC5924379; doi:10.2196/medinform.7744)
Supplement: Multimedia Appendix 1 [file medinform_v6i2e20_app1.pdf]

Without loss of generality, let us instantiate the objective function with the regularizer λ of empirical error on the labeled data for a family of hash codes.

$f(W, Q) = ¿$

$$min \sum_{i} \sum_{k} \left\| W_k^T P_k^i - H_k^i \right\|_F^2 + \lambda \sum_{i} \sum_{k} \sum_{u,v} -s_{k_{uv}}^i r_{uv}^i \quad \text{(A-1)}$$

One can express the second term of the above objective function in a compact matrix form by defining matrices $S_k^i \in R^{N_i \times N_i}$ and $R^i \in R^{N_i \times N_i}$ incorporating the pairwise similarity of P_k^i and the pairwise relationship of P^i for labeled information respectively. Then, $S\left(\{W_k\}_{k=1}^K\right)$ can be represented as

$$S\left(\{W_k\}_{k=1}^K\right) = \lambda \sum_{i} \sum_{k} tr\left(-S_k^i R^i\right) \quad \text{(A-2)}$$

and using (1) and absorbing the constant term into λ we can also represent it as

$$S\left(\{W_k\}_{k=1}^K\right) = \lambda \sum_{i} \sum_{k} tr\left(-H_k^i R^i H_k^{i\,T}\right). \quad \text{(A-3)}$$

The objective function becomes the follows.

$$f(W, Q) = min \sum_{i} \sum_{k} \left\| W_k^T P_k^i - sign\left(Q_k^i\right) \right\|_F^2 + ¿$$

$$\lambda \sum_{i} \sum_{k} tr\left(-sign\left(Q_k^i\right) R^i sign\left(Q_k^i\right)^T\right) \quad \text{(A-4)}$$

Here, we concern two possible problems due to the sign function for Q. First, Q may not be a unique solution and thus the objective function is difficult to converge without considering any regularizer about Q. We add a Frobenius norm regularizer η. In addition, the objective function $f(W, Q)$ is nondifferentiable in terms of Q. We can approximate the sign function with the surrogate function (A-5).

$$S_L\left(Q_k^i\right) = \left(Q_k^i \circ Q_k^i + \xi\right)^{\frac{-1}{2}} \circ Q_k^i \quad \text{(A-5)}$$

Here, ξ is a positive constant close to zero and $\circ$ is the hadamard (elementwise) product. Finally, we have the following objective function.

$$f(W, Q) = min \sum_{i} \sum_{k} \left\| W_k^T P_k^i - S_L\left(Q_k^i\right) \right\|_F^2 + ¿$$

$$\lambda \sum_i \sum_k tr\left(-S_L\left(Q_k^i\right) R^i S_L\left(Q_k^i\right)^T\right) + \eta \sum_i \sum_k \left\|Q_k^i\right\|_F^2 \quad \text{(A-6)}$$

To minimize the objective function applying (A-6), we use a Newton-Raphson algorithm [55] and iteratively solve W and Q. When updating W with Q fixed we consider the first derivative of W in terms of k without loss of generality.

$$\frac{\partial f}{\partial W_k} = \sum_j 2P_k^i \{W_k^T P_k^i - \left(Q_k^i \circ Q_k^i + \xi\right)^{\frac{-1}{2}} \circ Q_k^i \quad \text{(A-7)}$$

This approach allows us to update W_k for all $k(1 \le k \le K)$ simultaneously $W_k^{new} = W_k - \left(\frac{\partial^2 f}{\partial W_k^2}\right)^{-1} \frac{\partial f}{\partial W_k}$. Similarly with W fixed we can update Q. To be specific, we update Q_k^i for all combinations of (i,k), $1 \le i \le M$, $1 \le k \le K$ at the same time $Q_k^{i,new} = Q_k^i - \left(\frac{\partial^2 f}{\partial Q_k^{i\,2}}\right)^{-1} \frac{\partial f}{\partial Q_k^i}$. The first derivative of Q_k^i is

$$\frac{\partial f}{\partial Q_k^i} = -2\left\{W_k^T P_k^i - S_L\left(Q_k^i\right)\right\} \circ S_L{}'\left(Q_k^i\right) - \lambda \nabla tr + 2\eta Q_k^i \quad \text{(A-8)}$$

where ∇tr is defined in an elementwise manner

$$(\nabla tr)_{p,q} = S_L^{'}\left(Q_k^i; p, q\right)\left\{\left(\sum_{t=1}^{N_i} S_L\left(Q_k^i; t, q\right) R_{t,p}^i\right) + S_L\left(Q_k^i; p, q\right)\right\} \quad \text{(A-9)}$$

and $S_L^{'}(Q_k^i) = \left(Q_k^i \circ Q_k^i + \xi\right)^{\frac{-3}{2}}$. Moreover, we calculate second derivatives for the Hessian matrix. We first consider the W_k

$$\frac{\partial^2 f}{\partial W_k^2} = \sum_i 2P_k^i P_k^{i\,T} \quad \text{(A-10)}$$

In terms of Q_k^i, $\frac{\partial^2 f}{\partial Q_k^{i\,2}}$ is derived as

$$\left(\left[-2\left(W_k^T P_k^i\right) S_L^{''}\left(Q_k^i\right) + 2\left\{S_L{}'\left(Q_k^i\right) \circ S_L^{'}\left(Q_k^i\right) + S_L\left(Q_k^i\right) \circ S_L^{''}\left(Q_k^i\right)\right\}\right]\right)\left(\vec{I}_{(N_i b_k)}\right)^T - \lambda \nabla^2 tr + 2\eta I_{(N_i b_k)} \quad \text{(A-11)}$$

Where

$$\nabla^2 tr = \left[I_{(b_k)} \otimes \left\{ \left(J_{(N_i)} + I_{(N_i)} \right) \circ R^i \right\} \right] \circ$$

$$\left\{ \left(S_L^{'} \left(Q_k^i \right) \right) \left(S_L^{'} \left(Q_k^i \right) \right)^T \right\} + diag\left(\left(\vec{A} \right) \right) \quad . \qquad \text{(A-12)}$$

Here, $\otimes$ is the Kronecker product [59] and ¿ and $diag$ are to transform a matrix to a vector and a vector to a diagonal matrix respectively. In addition, J is a matrix of ones and A is defined in an elementwise manner as follows

$$(A)_{p,q} = S_L^{''}\left(Q_k^i; p, q\right) \left\{ \left(\sum_{t=1}^{N_i} S_L\left(Q_k^i; t, q\right) R_{t,p}^i \right) + S_L\left(Q_k^i; p, q\right) \right\} \qquad \text{(A-13)}$$

where $S_L^{''}(Q_k^i; p, q) = (Q_k^i \circ Q_k^i + \xi) - \frac{5}{2} Q_k^i$.
