# Supplementary material for: Privacy-Preserving Patient Similarity Learning in a Federated Environment: Development and Analysis
Source: JMIR Med Inform. 2018 Apr 13;6(2):e20. doi: 10.2196/medinform.7744 (PMC5924379; doi:10.2196/medinform.7744)
Supplement: Multimedia Appendix 2 [file medinform_v6i2e20_app2.pdf]

Table S1. Average AUC with standard deviation (parenthesis) of κ-NN (κ=1) from our system, open system and closed system

		Multi-hash			Uni-hash		Baseline		
Disease	Distance	Our system	Open system	Closed system	Open system	Closed system	Distance	Open system	Closed system
Disorders of lipoid metabolism	Hamming	0.9084 (0.0142)	0.9103 (0.0160)	0.8805 (0.0328)	0.9159 (0.0168)	0.8429 (0.0276)	Euclidean	0.5297 (0.0158)	0.5160 (0.0089)
							Cityblock	0.5268 (0.0115)	0.5216 (0.0108)
							Cosine	0.7116 (0.0312)	0.6866 (0.0297)
							Correlation	0.7070 (0.0301)	0.6800 (0.0294)
Hypertensive chronic kidney disease	Hamming	0.8816 (0.0422)	0.9114 (0.0440)	0.8312 (0.0466)	0.9270 (0.0144)	0.8455 (0.0280)	Euclidean	0.5227 (0.0124)	0.5141 (0.0136)
							Cityblock	0.5225 (0.0138)	0.5295 (0.0190)
							Cosine	0.6873 (0.0193)	0.6781 (0.0224)
							Correlation	0.6871 (0.0204)	0.6757 (0.0221)
Cardiac dysrhythmias	Hamming	0.8807 (0.0290)	0.9183 (0.0444)	0.8633 (0.0390)	0.9072 (0.0092)	0.8181 (0.0427)	Euclidean	0.5324 (0.0146)	0.5230 (0.0112)
							Cityblock	0.5290 (0.0127)	0.5302 (0.0138)
							Cosine	0.6866 (0.0158)	0.6562 (0.0254)
							Correlation	0.6807 (0.0157)	0.6548 (0.0220)
Heart failure	Hamming	0.8786 (0.0309)	0.9171 (0.0327)	0.8594 (0.0420)	0.9089 (0.0182)	0.8414 (0.0331)	Euclidean	0.5315 (0.0106)	0.5191 (0.0127)
							Cityblock	0.5312 (0.0153)	0.5293 (0.0154)
							Cosine	0.6923 (0.0350)	0.6712 (0.0367)
							Correlation	0.6907 (0.0341)	0.6689 (0.0370)
Acute renal failure	Hamming	0.8848 (0.0467)	0.9215 (0.0411)	0.8377 (0.0332)	0.8821 (0.0135)	0.7839 (0.0418)	Euclidean	0.5590 (0.0419)	0.5326 (0.0224)
							Cityblock	0.5737 (0.0390)	0.5516 (0.0252)
							Cosine	0.6609 (0.0335)	0.6408 (0.0277)
							Correlation	0.6603 (0.0347)	0.6423 (0.0268)

Table S2. Average AUC with standard deviation (parenthesis) of κ-NN (κ=3) from our system, open system and closed system

		Multi-hash			Uni-hash		Baseline		
Disease	Distance	Our system	Open system	Closed system	Open system	Closed system	Distance	Open system	Closed system
Disorders of lipoid metabolism	Hamming	0.9330 (0.0086)	0.9343 (0.0125)	0.9002 (0.0285)	0.9159 (0.0168)	0.8486 (0.0271)	Euclidean	0.5329 (0.0148)	0.5361 (0.0167)
							Cityblock	0.5341 (0.0121)	0.5639 (0.0269)
							Cosine	0.8079 (0.0222)	0.7945 (0.0308)
							Correlation	0.8043 (0.0244)	0.7824 (0.0299)
Hypertensive chronic kidney disease	Hamming	0.9078 (0.0346)	0.9283 (0.0432)	0.8538 (0.0421)	0.9270 (0.0144)	0.8501 (0.0305)	Euclidean	0.5506 (0.0325)	0.5584 (0.0323)
							Cityblock	0.5727 (0.0404)	0.5762 (0.0311)
							Cosine	0.7823 (0.0261)	0.7762 (0.0262)
							Correlation	0.7807 (0.0272)	0.7718 (0.0300)
Cardiac dysrhythmias	Hamming	0.9135 (0.0287)	0.9368 (0.0492)	0.8833 (0.0397)	0.9072 (0.0092)	0.8236 (0.0328)	Euclidean	0.5378 (0.0230)	0.5319 (0.0171)
							Cityblock	0.5456 (0.0225)	0.5500 (0.0237)
							Cosine	0.7695 (0.0151)	0.7340 (0.0343)
							Correlation	0.7660 (0.0144)	0.7284 (0.0320)
Heart failure	Hamming	0.9058 (0.0282)	0.9351 (0.0326)	0.8798 (0.0414)	0.9089 (0.0182)	0.8471 (0.0248)	Euclidean	0.5452 (0.0209)	0.5357 (0.0182)
							Cityblock	0.5534 (0.0281)	0.5462 (0.0235)
							Cosine	0.7986 (0.0292)	0.7733 (0.0421)
							Correlation	0.7949 (0.0281)	0.7643 (0.0415)
Acute renal failure	Hamming	0.9169 (0.0397)	0.9477 (0.0374)	0.8637 (0.0320)	0.8821 (0.0135)	0.7929 (0.0378)	Euclidean	0.5632 (0.0437)	0.5447 (0.0313)
							Cityblock	0.5958 (0.0355)	0.5721 (0.0291)
							Cosine	0.7434 (0.0380)	0.7289 (0.0341)
							Correlation	0.7427 (0.0380)	0.7281 (0.0347)

Table S3. Average AUC with standard deviation (parenthesis) of κ-NN (κ=9) from our system, open system and closed system

		Multi-hash			Uni-hash		Baseline		
Disease	Distance	Our system	Open system	Closed system	Open system	Closed system	Distance	Open system	Closed system
Disorders of lipoid metabolism	Hamming	0.9458 (0.0074)	0.9482 (0.0175)	0.9204 (0.0239)	0.9159 (0.0168)	0.8524 (0.0259)	Euclidean	0.5643 (0.0198)	0.5687 (0.0334)
							Cityblock	0.6116 (0.0360)	0.6162 (0.0336)
							Cosine	0.8795 (0.0225)	0.8596 (0.0232)
							Correlation	0.8745 (0.0227)	0.8489 (0.0248)
Hypertensive chronic kidney disease	Hamming	0.9278 (0.0332)	0.9409 (0.0322)	0.8710 (0.0371)	0.9270 (0.0144)	0.8555 (0.0302)	Euclidean	0.6554 (0.0503)	0.6199 (0.0320)
							Cityblock	0.6906 (0.0457)	0.6798 (0.0435)
							Cosine	0.8724 (0.0176)	0.8536 (0.0235)
							Correlation	0.8683 (0.0218)	0.8498 (0.0250)
Cardiac dysrhythmias	Hamming	0.9330 (0.0250)	0.9433 (0.0461)	0.9042 (0.0361)	0.9072 (0.0092)	0.8290 (0.0423)	Euclidean	0.5705 (0.0262)	0.5400 (0.0190)
							Cityblock	0.5913 (0.0355)	0.5913 (0.0323)
							Cosine	0.8383 (0.0170)	0.8064 (0.0236)
							Correlation	0.8327 (0.0131)	0.7968 (0.0228)
Heart failure	Hamming	0.9273 (0.0207)	0.9464 (0.0320)	0.9006 (0.0395)	0.9089 (0.0182)	0.8549 (0.0335)	Euclidean	0.5614 (0.0475)	0.5607 (0.0309)
							Cityblock	0.6145 (0.0562)	0.6062 (0.0586)
							Cosine	0.8620 (0.0310)	0.8304 (0.0338)
							Correlation	0.8587 (0.0305)	0.8239 (0.0344)
Acute renal failure	Hamming	0.9342 (0.0370)	0.9568 (0.0360)	0.8843 (0.0308)	0.8821 (0.0135)	0.7982 (0.0375)	Euclidean	0.5890 (0.0621)	0.5695 (0.0327)
							Cityblock	0.6235 (0.0700)	0.6198 (0.0407)
							Cosine	0.8222 (0.0254)	0.8012 (0.0271)
							Correlation	0.8230 (0.0260)	0.8002 (0.0279)
