# Supplementary material for: Privacy-Preserving Patient Similarity Learning in a Federated Environment: Development and Analysis
Source: JMIR Med Inform. 2018 Apr 13;6(2):e20. doi: 10.2196/medinform.7744 (PMC5924379; doi:10.2196/medinform.7744)
Supplement: Multimedia Appendix 3 [file medinform_v6i2e20_app3.pdf]

Disease	Multi-hash (Hamming distance)			Baseline (Cosine distance)	
	Our system	Open system	Closed system	Open system	Closed system
			F1		
Disorders of lipoid metabolism	0.6433 (0.0345)	0.7111 (0.0277)	0.6207 (0.0165)	0.5878 (0.0158)	0.5238 (0.0189)
Hypertensive chronic kidney disease	0.5967 (0.0344)	0.6167 (0.0217)	0.5730 (0.0201)	0.5403 (0.0168)	0.5063 (0.0202)
Cardiac dysrhythmias	0.6521 (0.0368)	0.6934 (0.0234)	0.6249 (0.0202)	0.6230 (0.0177)	0.5417 (0.0203)
Heart failure	0.6887 (0.0318)	0.7418 (0.0251)	0.6235 (0.0218)	0.6065 (0.0189)	0.5445 (0.0211)
Acute renal failure	0.6854 (0.0310)	0.7534 (0.0257)	0.6359 (0.0197)	0.5556 (0.0165)	0.5298 (0.0219)
			Sensitivity		
Disorders of lipoid metabolism	0.8263 (0.0214)	0.8374 (0.0278)	0.8302 (0.0198)	0.5918 (0.0198)	0.5391 (0.0187)
Hypertensive chronic kidney disease	0.8594 (0.0261)	0.8656 (0.0265)	0.7969 (0.0188)	0.4453 (0.0177)	0.4646 (0.0165)
Cardiac dysrhythmias	0.7862 (0.0289)	0.8397 (0.0366)	0.7862 (0.0205)	0.6512 (0.0165)	0.5446 (0.0178)
Heart failure	0.9248 (0.0335)	0.9854 (0.0331)	0.8195 (0.0277)	0.6131 (0.0204)	0.5412 (0.0154)
Acute renal failure	0.8714 (0.0368)	0.9929 (0.0311)	0.8618 (0.0267)	0.5357 (0.0211)	0.4834 (0.0201)
			Specificity		
Disorders of lipoid metabolism	0.7660 (0.0305)	0.7671 (0.0250)	0.6824 (0.0207)	0.8050 (0.0198)	0.7744 (0.0187)
Hypertensive chronic kidney disease	0.6261 (0.0312)	0.6746 (0.0291)	0.6061 (0.0211)	0.9228 (0.0178)	0.8327 (0.0154)
Cardiac dysrhythmias	0.7313 (0.0304)	0.7442 (0.0270)	0.6964 (0.0235)	0.7778 (0.0167)	0.8101 (0.0166)
Heart failure	0.6896 (0.0278)	0.7195 (0.0258)	0.6689 (0.0165)	0.8293 (0.0165)	0.8188 (0.0154)
Acute renal failure	0.7108 (0.0259)	0.7231 (0.0216)	0.6312 (0.0188)	0.8308 (0.0185)	0.8606 (0.0178)
